# Light-harvesting complexes of *Botryococcus braunii*

**DOI:** 10.1007/s11120-017-0405-8

**Published:** 2017-05-27

**Authors:** Tomas E. van den Berg, Bart van Oort, Roberta Croce

**Affiliations:** Biophysics of Photosynthesis, Department of Physics and Astronomy, Faculty of Sciences, VU University Amsterdam, 1081 HV Amsterdam, The Netherlands

**Keywords:** *Botryococcus braunii*, LHC, Light-harvesting, Loroxanthin, Pigment-protein complexes

## Abstract

The colonial green alga *Botryococcus braunii* (*BB*) is a potential source of biofuel due to its natural high hydrocarbon content. Unfortunately, its slow growth limits its biotechnological potential. Understanding its photosynthetic machinery could help to identify possible growth limitations. Here, we present the first study on *BB* light-harvesting complexes (LHCs). We purified two LHC fractions containing the complexes in monomeric and trimeric form. Both fractions contained at least two proteins with molecular weight (MW) around 25 kDa. The chlorophyll composition is similar to that of the LHCII of plants; in contrast, the main xanthophyll is loroxanthin, which substitutes lutein in most binding sites. Circular dichroism and 77 K absorption spectra lack typical differences between monomeric and trimeric complexes, suggesting that intermonomer interactions do not play a role in *BB* LHCs. This is in agreement with the low stability of the *BB* LHCII trimers as compared to the complexes of plants, which could be related to loroxanthin binding in the central (L1 and L2) binding sites. The properties of *BB* LHCII are similar to those of plant LHCII, indicating a similar pigment organization. Differences are a higher content of red chlorophyll *a*, similar to plant Lhcb3. These differences and the different Xan composition had no effect on excitation energy transfer or fluorescence lifetimes, which were similar to plant LHCII.

**Electronic supplementary material:**

The online version of this article (doi:10.1007/s11120-017-0405-8) contains supplementary material, which is available to authorized users.

## Introduction

*Botryococcus braunii* (*BB*) Kützing (*Trebouxiophyceae*, Chlorophyta) is a colonial green alga found in fresh and brackish lakes and ponds throughout different climate zones with big potential for production of biofuel, due to its natural high hydrocarbon content (Metzger and Largeau 2005) and in some strains of carbohydrates with industrial potential (Fernandes et al. 1989). However, application is severely limited by its slow growth in culture, despite the occurrence of natural blooms (Wake and Hillen 1980). Strategies for improvements are hampered by the limited knowledge on genome and physiology. Photosynthesis and its regulation have received little attention in *BB* research. In this study, we make a start towards understanding the primary processes of *BB* photosynthesis by characterizing its light-harvesting complexes (LHCs).

LHCs are protein complexes densely packed with pigments. Their role is to increase the absorption cross section of the photosystems, which convert light energy into chemical energy. LHCs of green plants and algae belong to a large gene family. They are integral membrane proteins composed of an apoprotein of 22–30 kDa that binds Chlorophyll (Chl) *a,b* and carotenoids (Jansson 1999; Ballottari et al. 2012; Büchel 2015). The main antenna complex of plants and algae, LHCII, is the product of several genes encoding highly homologous proteins and it occurs primarily in trimeric form. LHCII binds 14 Chls (8 Chls *a* and 6 Chls *b*) and four xanthophylls (Xan), and is well conserved throughout plants and green algae with only minor differences in Xan content (Durnford et al. 1999). Plant LHCII binds two luteins (Lut), one neoxanthin (Neo), and one violaxanthin (Vio) (Liu et al. 2004) opposed to LHCII of the model alga *Chlamydomonas reinhardtii* (*CR*), where lutein is partially replaced by loroxanthin (Loro) (Natali and Croce 2015).

In addition to LHCII, several other LHCs are present in plants and algae as monomers mainly associated with PSII or dimers associated with PSI. These complexes bind 13–14 Chls in most plants and generally have a higher Chl *a*/*b* ratio than LHCII (Ballottari et al. 2012).

LHCs are not only important for light harvesting but also essential for photoprotection under excess light conditions, in short term through non-photochemical quenching (NPQ) (Niyogi and Truong 2013) and in long term because by light-dependent regulation of their number relative to the photosystems (antenna size) (Kouřil et al. 2013).

Here, we studied *BB* strain CCALA 778 (subclade 5, race-A), which has a particularly high potential for applications in different industries (Gouveia et al. 2017). We have developed protocols to purify its LHCs, and characterized them by spectroscopic and biochemical methods. The properties of *BB* LHCs are compared to those of the LHC complexes of vascular plants and of the green alga *C. reinhardtii*.

## Materials and methods

### Strain, cultivation conditions, and thylakoids preparation

*Botryococcus braunii* CCALA778 (subclade 5, race-A) was obtained from the culture collection of autotrophic organisms (Institute of Botany, Academy of sciences of the Czech Republic). Cultures were inoculated from a stock culture at a dry weight of ~0.1 g L^−1^ and cultured in 500 mL mCHU-13 (3xN) (Grung and Metzger 1989) in a 1-L shake flask with bubbling with air enriched with 5% CO_2_. Shaking was 170 rpm; the light intensity was 50 µE m^2^ s^−1^ from a white fluorescent tube, and temperature was 25 °C. Cultures were harvested after 2 weeks before the onset of the stationary phase. Thylakoids were prepared as in (Chua 1975) with addition of 1 mM benzamidine and ε-aminocaproic acid to the disruption buffer.

### Isolation of LHCs

Thylakoids (kept in the dark on ice) at a Chl concentration of 0.5 mg mL^−1^ were mixed with an equal volume of freshly prepared detergent in buffer and allowed to solubilize for 20 min at 0, 4 °C or room temperature in the dark with end over end shaking. Dodecyl-β-d-maltoside (β-DM) (Anatrace), dodecyl-α-d-maltoside (α-DM) (Anatrace) and octyl-β-d-glucoside (OG) (Anatrace) at concentrations ranging from 0.5 to 2% (w/w) in steps of 0.25% were used. Isolation of complexes was performed by sucrose density gradient as described in (Drop et al. 2014), while varying the detergent nature and concentration. A 2 M sucrose cushion was used for visual inspection of unsolubilized material.

### Protein analysis

SDS-PAGE was performed with a tris-tricine buffer system (Schägger 2006) with 14% acrylamide and 6 M urea in the gel. Sample loading was 1.5 µg Chl for monomeric and trimeric fractions and 3 µg for thylakoids.

Western blot was performed with a mini-blot module. Protein transfer was checked with Ponceau staining. Washed nitrocellulose membranes were incubated for 1 h at room temperature (RT) with primary antibody at the recommended concentration by the manufacturer (Agrisera). Antibodies used were Lhcb3 (AS01002), Lhcb2 (AS01003), Lhcb1 (AS01004), Lhcb5 (AS01009), Lhcb6 (AS01010), Lhcb4 (AS04045), Lhcb4 (*CR)* (AS06117), and Lhcb5 (*CR)* (AS09407). Secondary antibody (goat anti rabbit, AS09602) was used at a 1:10,000 dilution and incubated for 1 h at RT. Visualization was done with a chemiluminescence assay and images were made with an LAS-4000 system (GE healthcare).

### Pigment analysis

The pigment composition of the fractions was determined by fitting the absorption spectrum of the 80% acetone extracts with the spectra of the individual pigments in the same solvent as described in (Croce et al. 2002a) and by HPLC. HPLC was performed on a System gold 126 equipped with a 168 UV–VIS detector (Beckman Coulter, USA) using a reverse phase C_18_-Sphereclone column (Phenomenex 5U ODS1, 00G-4143-E0, 4.6 mm × 250 mm) according to the protocol in (Pineau et al. 2001). The chromatogram together with the spectrum of Loro in 80% acetone/dioxane is plotted in online resource 1.

### Absorption, fluorescence, and circular dichroism

The sample buffer for all RT experiments was 0.5 M sucrose, 20 mM Tricine (pH 7.8), 0.06% α-DM. In addition for 77 K experiments, the buffer contained 66% (w/w) glycerol. Sample OD at the maximum in the Qy region was 0.8–1 for absorption and CD and less than 0.05 for fluorescence measurements.

Room temperature and 77 K absorption spectra were recorded with Cary 4000 spectrophotometer (Varian) with a spectral bandwidth of 2 nm. 77 K absorption spectra were measured with a home-build liquid N_2_-cooled low-temperature device in the same spectrophotometer. Fluorescence emission and excitation spectra at RT and 77 K were recorded on a Fluorlog 3.22 spectroflorimeter (Jobin-Yvon spex). Samples were cooled in a cryostat (Oxford Instruments) for 77 K measurements. For fluorescence emission spectra, the spectral bandwidths were 3 nm for excitation, and 1 nm for emission. For fluorescence excitation spectra, the spectral bandwidths were 1 nm for excitation and 5 nm for emission. CD spectra were recorded at 20 °C with a Chirascan CD spectrophotometer (Applied Photophysics) equipped with a temperature control unit TC125 (Quantum Northwest). The spectral bandwidth was 1 nm. For temperature stability measurements, the sample holder temperature was increased from 10 to 85 °C in steps of 5 °C. Each heating step took 1 min, followed by 4 min of equilibration before measuring the CD at 682 nm. Each temperature point represents the average of 100 data points of 1.05 s integration time.

### Time-resolved fluorescence

Time-resolved fluorescence was measured at RT by time-correlated single photon counting (TCSPC) with a FluoTime 200 fluorometer (PicoQuant). Samples were stirred with a magnetic stirring bar in a 1 cm quartz cuvet. Excitation was with a laser diode at 468 nm, with 5 MHz repetition rate and 1 µW power. Careful checks at higher and lower power confirmed the absence of non-linear processes (e.g., annihilation). Fluorescence was detected with 4 ps timesteps, at 682 nm (8 nm bandwidth), at an angle of 90° with the excitation, through a polarizer set at magic angle relative to the excitation polarization. The instrument response function (FWHM 88 ps) was determined with the emission of pinacyanol iodide in methanol (6 ps lifetime (van Oort et al. 2008)). Data were accumulated until the number of counts in the peak channel was 20,000. Fluorescence decay curves were fitted with a multi-exponential decay ($F\left( t \right)=\mathop \sum \nolimits_i {A_i}{e^{ - t/{\tau _i}}}$, with amplitudes ${A_i}$ and lifetimes ${\tau _i})$ convoluted with the IRF with the Fluofit software (PicoQuant). Three components were necessary to get a good fit of the data as judged by χ^2^, the distribution of the residuals around 0 and the autocorrelation function of the residuals. Average lifetimes are calculated as $~{\tau _{{avg}}}={{\mathop \sum \limits_i {A_i}{\tau _i}} \mathord{\left/ {\vphantom {{\mathop \sum \limits_i {A_i}{\tau _i}} {\mathop \sum \limits_i {A_i}}}} \right. \kern-\nulldelimiterspace} {\mathop \sum \limits_i {A_i}}}$.

## Results

### Isolation of LHCs

**Fig. 1:**
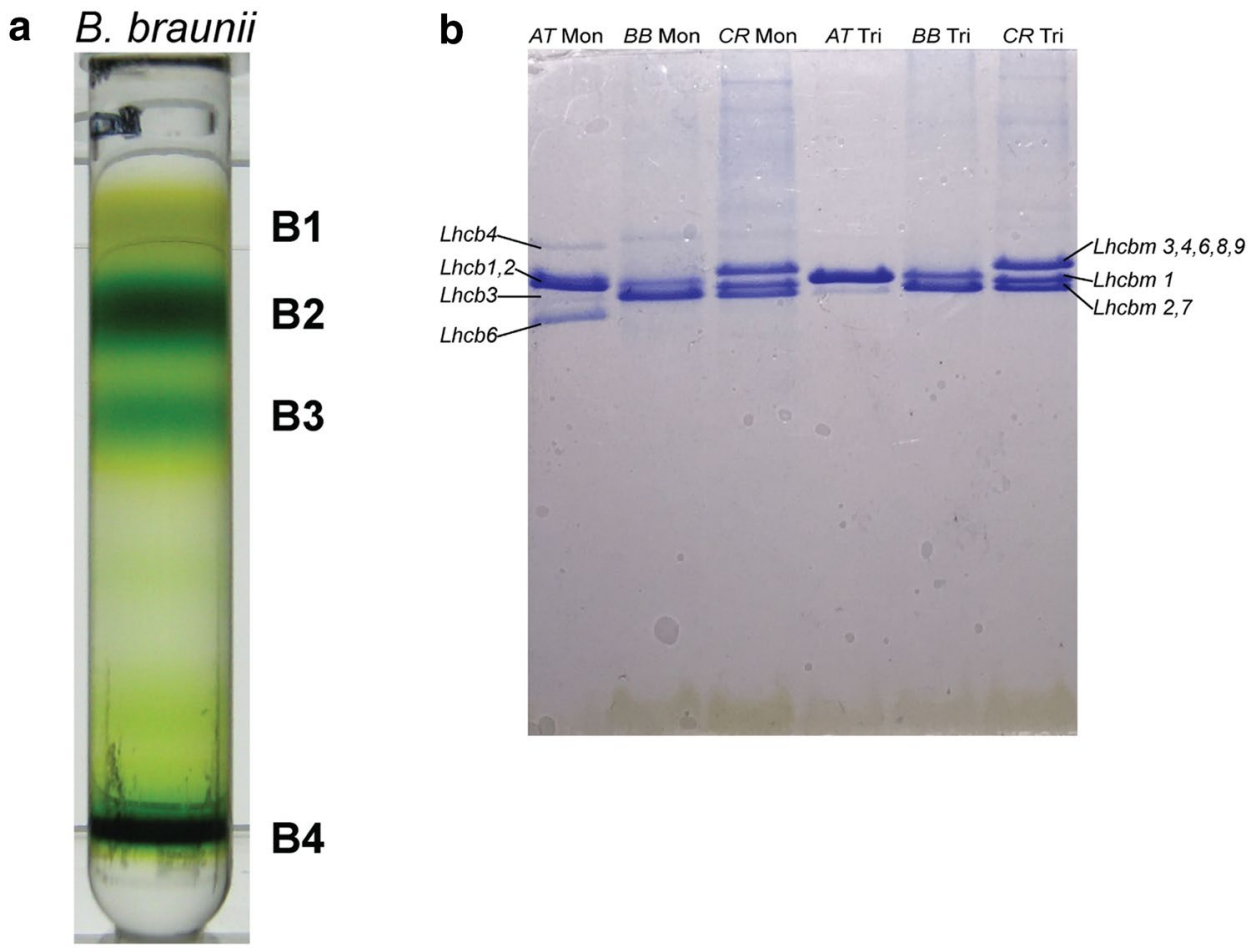
Isolation of LHCs. **a** Separation of complexes from solubillized *BB* thylakoids (1% α-DM) by sucrose density gradient ultracentrifugation. *B1* free pigments, *B2* monomeric fraction, *B3* trimeric fraction, *B4* unsolubilized material. **b** Tricine SDS-PAGE of the monomeric and trimeric fractions, together with monomeric and trimeric LHC of *C. reinhardtii* and *A. thaliana*. Equal amounts of Chl were loaded in each lane. (Mon) monomeric fraction, (Tri) trimeric fraction

The thylakoid membranes isolated from *B. braunii* proved more difficult to solubilize than their counterparts in *C. reinhardtii* or plant. Different detergents (β-DM, α-DM, and OG), different ratios of detergent to Chl concentration, and different solubilization temperatures were tested to optimize the yield of LHCs. The solubilized membranes were separated by ultracentrifugation in sucrose density gradients. α-DM and OG (OG is not shown) solubilization resulted in two main green bands in the sucrose gradient at MW corresponding to monomeric and trimeric LHCs (Fig. 1a, B2–B3). Additional bands consisted of free pigments (B1) and unsolubilized material (B4). The SDS-PAGE shows that bands B2 and B3 contain at least two proteins with a molecular weight in the range of that of the LHCs of *A. thaliana* and *C. reinhardtii* (Fig. 1b). Minor differences between the monomeric and trimeric fractions in the SDS-PAGE are probably due to the presence of minor antennae in B2, similar to Lhcb4, b5, and b6 of *A. thaliana* (Caffarri et al. 2009). After β-DM solubilization, the lower trimeric gradient band was very faint (online resource 2), suggesting that the trimers are less stable than in vascular plants, where they are retained during β-DM solubilization (Ruban et al. 1999).

At variance with plants (Xu et al. 2015) and *C. reinhardtii* (Drop et al. 2014), where bands containing PSI and PSII (super)complexes are also present in the gradient upon solubilization of the thylakoids with α-DM, no other green bands were observed in *B. braunii*. This suggests that this detergent concentration does not extract all the complexes from the membrane. Indeed, an intense PSI band was observed using stronger (1% β-DM) solubilization conditions (online resource 2), while PSII supercomplexes could not be purified, probably because they dissociate in β-DM and are not extracted from the membrane in α-DM. For the purification of LHCs 1% α-DM at room temperature was selected as the optimal solubilization condition (Fig. 1a), because it is a good compromise between yield and sample quality: lower concentrations yielded considerably less complexes and higher concentrations could lead to pigment loss (Ruban et al. 1999).

Next we performed western blot analyses using antibodies that recognize LHC proteins of plants and *C. reinhardtii*. However, no bands were recognized in the *BB* fractions (online resource 3), indicating that the specific epitopes are not conserved.

### Pigment composition

The pigment compositions of the two green fractions are reported in Table 1 together with those of the same fractions of the LHCs of *A. thaliana* and *C. reinhardtii*. The Chl *a*/*b* value of the trimeric band (1.38) is very close to that of plants LHCII, which contains eight Chls *a* and six Chls *b* per monomer, suggesting a similar Chl composition. The slightly higher Chl *a*/*b* ratio of the monomers (1.47) is probably at least partially due to a small amount of minor antennae, which on average have a higher Chl *a*/*b* ratio in plants. Selective loss of Chl *b* during monomerization might also contribute. The Chl/carotenoid (Car) ratio is 4.8–5 for both fractions, thus much higher than in monomeric and trimeric LHCs of plants. This can be due to (i) *BB* LHCs bind more Chl or less Xan per monomer or (ii) some Xan are lost during purification. Four Xan species are associated with the *BB* complexes: Lutein (Lut), Violaxanthin (Vio), Neoxanthin (Neo), and Loroxanthin (Loro). The first three are also present in plants, and Loro in *C. reinhardtii*. The lower Lut content of *BB* LHCII suggests that the L1 and L2 binding sites are partly occupied by Loro, as in *C. reinhardtii* (Natali and Croce 2015). The lower Neo content is surprising, because Neo is generally strongly bound to the complexes and its binding site is well conserved and highly specific (Caffarri et al. 2007). The Neo binding site could be missing in some of the *BB* complexes, or have a weak binding affinity. Vio content is also low, but as this Xan in plants is mainly located in a weak binding site (Ruban et al. 1999; Caffarri et al. 2001), it can be lost during purification.

**Table 1:** Pigment composition

Sample	Chl *a*/*b*	Chl/car	Loro/mon	Neo/mon	Vio/mon	Lut/mon	Chl/mon
Monomers AT^a^	1.85 ± 0.01	3.65 ± 0.01	–	0.73 ± 0.01	0.71 ± 0.01	2.39 ± 0.02	14
Monomers CR^a^	1.29 ± 0.06	3.8 ± 0.5	1.4 ± 0.2^b^	1^b^	0.55 ± 0.07	0.91 ± 0.04	14
Monomers BB	1.47 ± 0.03	5.0 ± 0.2	1.5 ± 0.2	0.45 ± 0.08	0.34 ± 0.07	0.63 ± 0.05	14
Trimers AT^a^	1.44 ± 0.01	3.54 ± 0.01	–	1.01 ± 0.01	0.29 ± 0.01	2.65 ± 0.01	14
Trimers CR^a^	1.28 ± 0.02	3.6 ± 0.1	0.7 ± 0.2^b^	1^b^	0.68 ± 0.05	1.4 ± 0.2	14
Trimers BB	1.38 ± 0.07	4.8 ± 0.4	1.5 ± 0.3	0.43 ± 0.08	0.20 ± 0.07	0.63 ± 0.05	14

The data are the result of four repetitions on two biological replicas

^a^Values for *CR* and *AT* are from literature (Dall’Osto et al. 2007; Drop et al. 2014)

^b^Loro and Neo were not separated in the pigment analyses of *CR*, and were therefore estimated under the assumption of 1 Neo per monomer because the binding site is conserved (Natali and Croce 2015). Chl/monomer was fixed at 14

### Absorption

**Fig. 2:**
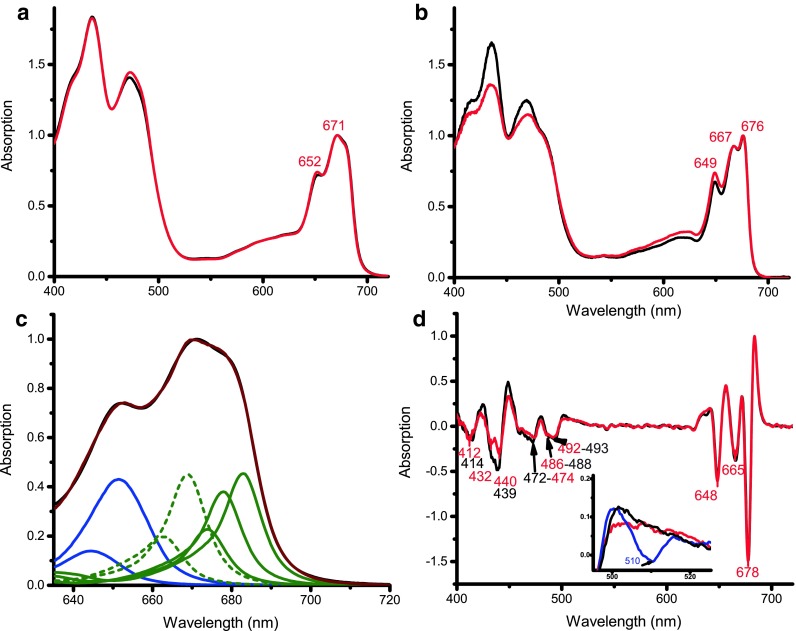
Absorption spectra, Q_Y_ fitting and second derivative of the monomeric and trimeric fractions. **a** RT absorption spectra normalized to the Q_Y_ maximum (monomers *black*, trimers *red*). **b** 77 K absorption spectra normalized to the Q_Y_ maximum (monomers *black*, trimers *red*). **c** Absorption spectrum of trimeric complexes fitted with the spectra of Chl *a* and Chl *b* in protein environment (Cinque et al. 2000). *Blue* represents Chl *b* spectral forms, *green* represents Chl *a* spectral forms (*solid*:* red *spectral forms, *dotted: blue* spectral forms). The measured spectrum is in *black* and the fitting result in *brown*. **d** Second derivative spectra of the 77 K absorption spectra normalized to the 684 nm maximum. In **a, b**, and **c** labels indicate the same peak positions in both fractions. In **d**, *black* is the monomeric fraction, *red* to trimeric, and *blue* (*inset*) to *AT* LHCII trimers

The absorption spectra of monomers and trimers are very similar both at RT (Fig. 2a) and 77 K (Fig. 2b) in agreement with their similar protein and pigment composition and they have the general features of LHCII from other species. The RT absorption spectra in the Q_Y_ region peak at 652 and 671 nm, with a shoulder at 682 nm (Fig. 2a). At 77 K the spectra peak at 649, 667, and 676 nm (Fig. 2b).

The maxima of both fractions at RT are 4 nm blue-shifted relative to plant LHCII, similar to *CR* (Drop et al. 2014). However, there are more Chls that are further red-shifted contributing to the 682 nm absorption.

To get more insights in the spectral forms contributing to the total absorption, the Q_y_ region of the spectrum of the trimer was fitted with the spectra of Chl *a* and Chl *b* in protein environment (Cinque et al. 2000) (Fig. 2c). Two Chls *b* forms (peaking at 644 nm, with an amplitude corresponding approximately to 1.5 Chls and at 651 nm, with amplitude 4.5) and 5 Chls *a* forms [663 (amplitude 1), 669 (2), 674 (1), 678 (2), and 683 nm (2)] were sufficient for a satisfactory description of the absorption spectrum. The analysis shows that the spectrum is composed of energetically well-separated pools of Chls *a* (blue and red). The spectral composition resembles that of Lhcb3 of plants (Caffarri et al. 2004) especially in the high amplitude of the red-most forms, with the main difference that a second Chl form is also red-shifted from 674 nm in Lhcb3 to 678 nm in *BB* LHCII.

In the Soret region, the second derivative of the 77 K spectrum shows minima at 439, 472, 488, and 493 nm for the monomers and at 440, 474, 486, and 492 nm for the trimers. Similar values were reported for plants LHCII (Caffarri et al. 2004). The most striking difference is the absence of the contribution around 510 nm (Ruban et al. 2000; Lampoura et al. 2002) in the trimeric fraction (blue curve in Fig. 2d inset). In plants, this red-shifted signal is due to the Lut in site L2 that twists upon trimerization (Ruban et al. 2000; Yan et al. 2007). The absence of this signal in *B. Braunii* can be due to the occupancy of the L2 site by loroxanthin.

### Circular dichroism (CD)

**Fig. 3:**
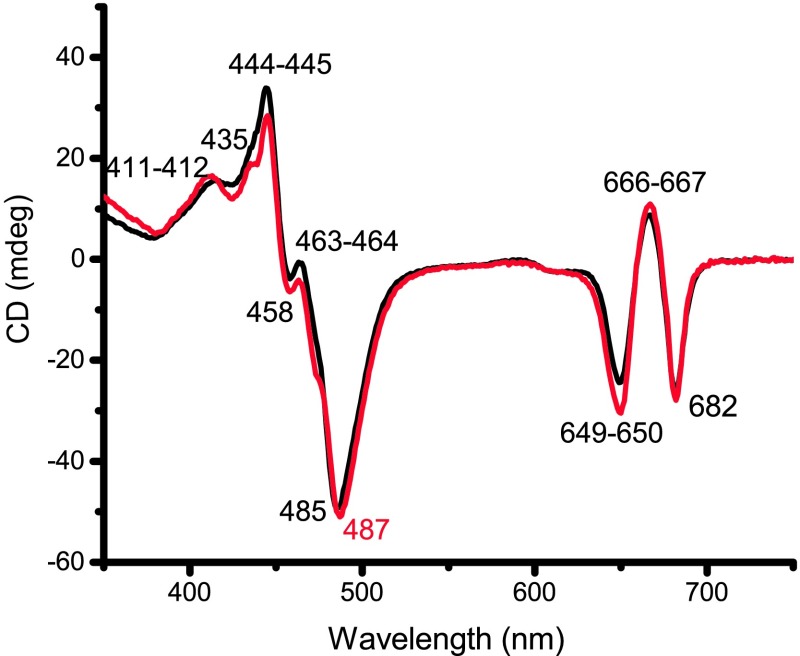
Circular dichroism (CD) spectra at RT of the monomeric (*black*) and the trimeric (*red*) fraction normalized to the Q_Y_ absorption maximum. *Labels* indicate peak positions. Only the ~487 nm peak position is different between the two samples

The CD spectra of the monomeric and trimeric fractions show only small differences (Fig. 3). This was also seen in *C. reinhardtii* (Natali and Croce 2015), but is at variance with plant LHCII, where the ratio of the amplitude of the negative bands at 470 and 490 nm is typically very different in monomers and trimers (Hobe et al. 1994; Georgakopoulou et al. 2007). To check if the spectral similarity of monomers and trimers in *B. Braunii* is caused by destabilization of trimeric LHCII during isolation, we also measured the CD spectrum of the solubilized thylakoid membranes (online resource 4) (Akhtar et al. 2015). No differences are observed between the spectrum (450–500 nm) of the membranes and that of LHCII suggesting that protein–protein interactions between monomeric units of the trimer do not influence the coupling between pigments.

In the Q_Y_ region, the spectrum displays the negative–positive–negative peak sequence typical of LHCII. However, the peaks are 1–3 nm blue-shifted compared to those in vascular plant trimers (Georgakopoulou et al. 2007; Akhtar et al. 2015), in line with the difference in absorption. In the Soret region, the main negative peak is 4–7 nm blue-shifted compared to plants (Georgakopoulou et al. 2007; Akhtar et al. 2015), possibly due to the differences in Chl–Xan interactions.

### Fluorescence emission spectra

**Fig. 4:**
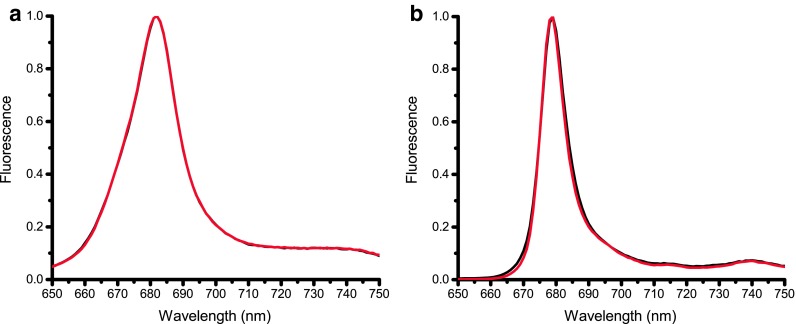
Fluorescence emission spectra of the monomeric (*black*) and trimeric (*red*) LHC fractions. **a** RT spectra normalized to the maximum. **b** 77 K spectra normalized to the maximum

The fluorescence emission spectra at RT and 77 K upon preferential Chl *a* excitation at 440 nm are the same for monomeric and trimeric fractions (Fig. 4). Preferential excitation of Chl *b* (475 nm) or Car (500 nm) yields the same normalized RT spectra (results not shown), indicating that the samples contained no disconnected Chls. At RT the spectra peak at 682 nm, which is 1 nm red-shifted relative to vascular plants and 4 nm relative to *CR* LHCII (Drop et al. 2014).

At 77 K the emission maximum of both fractions is blue-shifted to 679 nm, with a full width at half maximum (FWHM) of 9 nm similar to vascular plants (Hemelrijk et al. 1992; Palacios et al. 2002) and 2 nm red-shifted relative to *CR* LHCII (Drop et al. 2014; Natali and Croce 2015).

### Time-resolved fluorescence

**Fig. 5:**
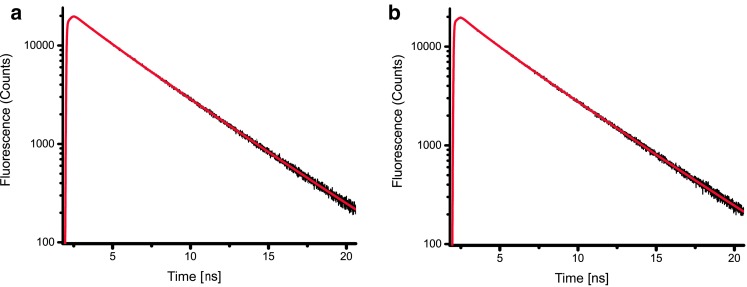
Fluorescence decay curves detected at 682 ± 4 nm. **a** Monomeric fraction, **b** trimeric fraction

Fluorescence decay traces of *BB* LHCs, measured by TCSPC, are shown in Fig. 5. The decay kinetics of the two fractions are very similar, and independent on the emission wavelength (online resource 5). The decays are well described with three components (Fig. 5; Table 2): a fast component of 0.3 ns with a small amplitude, an intermediate component of 2.2–2.4 ns with an amplitude of approximately 25–30% and a long component of 4.1 ns with an amplitude of approximately 60%. The average lifetime (*τ*_avg_) is 3.1 ns for the monomeric fraction and 3.2 ns for the trimeric fraction. This is very similar to the values reported for *CR* monomers and trimers (Natali and Croce 2015) and plant Lhcb3 monomers (Palacios et al. 2006), and slightly shorter than for trimeric LHCII of plant (Moya et al. 2001; van Oort et al. 2007).

**Table 2:** Parameters of the multi-exponential decay model used to fit the fluorescence decays

Sample	*τ*_avg_ (ns)	*A*_1_ (%)	*τ*_1_ (ns)	*A*_2_ (%)	*τ*_2_ (ns)	*A*_3_ (%)	*τ*_3_ (ns)
Monomers	3.2 ± 0.2	11 ± 1	0.29 ± 0.07	31 ± 11	2.4 ± 0.5	58 ± 9	4.1 ± 0.3
Trimers	3.1 ± 0.2	14 ± 3	0.31 ± 0.07	24 ± 9	2.2 ± 0.5	62 ± 7	4.1 ± 0.2

Errors represent the standard deviation of ≥3 biological replicas

*A* amplitude and *T* lifetime of the fitted fluorescence decay. *T*_*avg*_ is the average lifetime

### Fluorescence excitation spectra

**Fig. 6:**
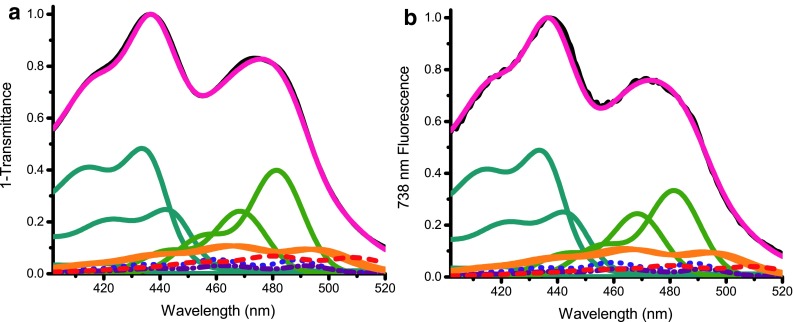
Spectral deconvolution of the 1-T and the excitation spectrum, detected at 738 nm, of the trimeric fraction in their individual pigment components (400–520 nm) **a** 1-T spectrum (fitted spectrum in *pink*). **b** Excitation spectrum (fitted spectrum in *pink*). The individual fitted components are indicated in color: *Blue-green* is Chl *a*, green Chl *b, orange* Loro, *red-dashed* Lut, *purple dash-dotted* Vio and *violet-dotted* Neo

To estimate the energy transfer efficiency between Xan and Chls in the trimeric fraction, we measured the fluorescence excitation spectrum. We fitted this spectrum with the spectra of the individual pigments, and compared this with the same analysis of the (1-T) spectrum, using the method described in (Croce et al. 2000) (Fig. 6). A satisfactory fit was obtained with 3 Chl *a*, 2 Chl *b*, 1 Neo, 1 Vio, 2 Loro, and 1 Lut (shifted) spectral forms. It was necessary to include a Lut form with its lowest energy transition peaking at 508 nm and with an amplitude representing 14% of the total carotenoids, although this transition was not clearly observed in the 77 K second derivative spectrum. The resulting excitation energy transfer (EET) efficiencies were on average 92% for Xan and 95% for Chl *b*. Chl *b* EET efficiency is expected to be 100% based on the absence of Chl *b* fluorescence emission upon excitation at 475 nm (see above); thus an error of 5% is assumed. The red-most Lut form had a low EET efficiency compared to the other Xan. These assignments and individual transfer efficiencies have to be handled with care due to the lack of data on the exact transition energies of the Xan in *BB*. The overall transfer efficiencies are high for LHCII (Caffarri et al. 2001, 2004; Das and Frank 2002).

### Thermal stability

**Fig. 7:**
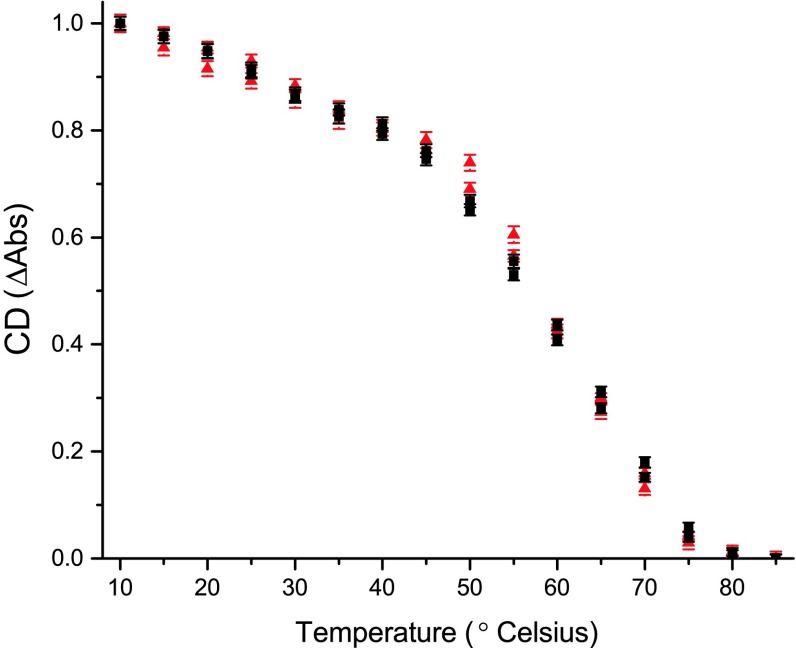
Thermal denaturation of the fractions followed by the loss of the 682 nm (−) CD signal at increasing temperature. *Black triangles* monomeric fraction, *red squares* trimeric fraction. Two biological replicas per fraction are plotted individually. *Error bars* represent the standard deviation of 100 technical replicas

The thermal stability of the complexes of both fractions was measured following the decrease of the 682 nm (−) CD signal with increasing temperature. The resulting curves (Fig. 7) are not single sigmoids as expected for protein denaturation (Greenfield 2009), but consist of a superposition of several components with different thermal stabilities, probably reflecting the heterogeneity of the preparation. The highest apparent transition, around 60 ± 2 °C, is between the values reported for *CR* monomers (Natali and Croce 2015) and *Pisum sativum* monomers (Zhang et al. 2012).

## Discussion

To obtain a good yield of solubilized membrane proteins from the *BB* thylakoids, we needed higher detergent:Chl ratios than in vascular plants or *C. reinhardtii*. It is unlikely that this is caused by co-purification of other protein-filled membranes, leading to a decreased detergent-to-protein/lipid ratio, because SDS-PAGE analyses of the thylakoids compared with those of higher plants loaded on a Chl basis showed no large differences (online resource 6). The differences in solubilization are probably due to the different composition of *BB* thylakoid membranes, which have higher DGDG/MGDG (di-/mono-galactosyldiacylglycerol) ratios (Moutel et al. 2016) than in plants (Sakurai et al. 2006) and *C. reinhardtii* (Vieler et al. 2007). DGDG and MGDG are only found in the chloroplast and make up the largest part of the thylakoid lipids. MGDG is a non-bilayer forming lipid that in pure form adopts an inverted hexagonal phase in aqueous medium (Shipley et al. 1973). In contrast, pure DGDG forms a L_α_ phase and promotes membrane stacking through hydrogen bonding between polar headgroups (Demé et al. 2014). Furthermore, DGDG regulates the formation of LHCII macroarrays in plant thylakoids (Krumova et al. 2010). Thus, a higher DGDG/MGDG ratio could make the thylakoids more detergent resistant because it decreases the disorder and makes the bilayer more tightly packed (Lichtenberg et al. 2005).

No PSII supercomplexes were observed under any tested solubilization condition. We conclude that the conditions required for the solubilization are such that LHC-PSII interactions are not retained, leading to disassembly of the supercomplexes. Moreover, the LHC monomeric fraction is larger than the trimeric fraction (Fig. 1a), while they have similar pigment and protein composition (Fig. 1b; Table 1). This indicates that the monomeric fraction mainly consists of trimers that have monomerized, as is observed in *C. reinhardtii* (Drop et al. 2014; Natali and Croce 2015). This implies that the trimers of *BB* are less stable than those of plants, where a similar detergent concentration does not break the monomer–monomer interactions to the extent observed here (Bielczynski et al. 2016). In line with weaker protein–protein interactions, the CD spectrum does not show the typical signature of the trimer, indicating that the trimerization does not change the spectroscopic properties of the individual monomers.

### Pigment composition

The Chl composition of *BB* LHCII is virtually identical to that of the complexes of plants and algae, suggesting that both the binding sites and their specificity are conserved. Instead the carotenoid composition differs, both in total amount relative to Chl and in the nature of the Xans. The Xan/Chl ratio is lower, which can be due to loss of Xan. Indeed the purification of LHCs often leads to the loss of pigments from peripheral binding sites (Ruban et al. 1999; Caffarri et al. 2001). However for LHCs of higher plants, increased solubilization strength leads to the loss of pigments in the following order: Vio, Lut, Neo (Ruban et al. 1999). If the binding sites in *BB* LHCII were conserved, as in higher plants LHCII, it would thus be unlikely that Neo/Chl would be reduced to the extend we observe here. This suggests that some of the complexes in the isolated fractions do not contain the Neo binding site, probably lacking the tyrosine that stabilizes the binding of Neo (Caffarri et al. 2007), as is observed for Lhcb6 (Passarini et al. 2009) and in all Lhca antennas (Croce et al. 2002a, b).

Lut, which is the main xanthophyll in LHCII of plants, is largely substituted by Loro (Table 1). In plant LHCII, Lut is associated with the two internal binding sites L1 and L2. In *BB* LHCII, these two sites are probably largely occupied by Loro. The data show that this substitution does not affect the energy transfer efficiency and fluorescence lifetimes. However, it can probably play a role in the lower stability of the trimers, since Lut is essential for the trimerization of plants LHCII (Lokstein et al. 2002; Havaux et al. 2004; Dall’Osto et al. 2006).

### Spectral signature

The CD features of *BB* LHCII are similar to those of LHCII of plants indicating that the pigment organization and thus the protein folding are conserved. The deconvolution of the absorption spectrum of *BB* LHCII with the spectra of individual Chls shows that most of the spectral forms are conserved with respect to plant LHCII, again indicating a similar environment for most of the pigments. There are also clear differences, such as the intense absorption at 683 nm, which in Lhcb1 and Lhcb2 of plants corresponds to the absorption of only one Chl. Its amplitude here is doubled, as in Lhcb3, suggesting that the LHCII of *BB* are more similar to this plant isoform. This is further supported by: (i) Lhcb3 is not able to form stable homotrimers, (Caffarri et al. 2004) (ii) Lhcb3 loses Neo more easily than the other Lhcbs (Caffarri et al. 2004; Palacios et al. 2006) and (iii) Lhcb3 has a slightly higher Chl *a*/*b* ratio (Palacios et al. 2006; Zhang et al. 2008).

### Could the lower trimer stability have a physiological function?

We show that trimeric LHCs in *BB* are not very stable, which suggests that they can monomerize easily also in vivo. In higher plants under HL, the amount of LHCII monomers was found to increase (Garab et al. 2002; Bielczynski et al. 2016). In addition, it was shown in vitro that monomeric LHCs are more susceptible to quenching than trimeric LHCs (Wentworth et al. 2000, 2004; Garab et al. 2002; van Oort et al. 2007). Thus, switching from a trimeric to a monomeric state could aid the quenching of these antennas in vivo (Garab et al. 2002).

## Electronic supplementary material

Below is the link to the electronic supplementary material.

Supplementary material 1 (PDF 357 KB)
