# Supplementary material for: Light-harvesting complexes of Botryococcus braunii
Source: Photosynth Res. 2017 May 27;135(1):191–201. doi: 10.1007/s11120-017-0405-8 (PMC5783996; doi:10.1007/s11120-017-0405-8)
Supplement: Supplementary file 1 — Supplementary material 1 (PDF 357 KB) [file 11120_2017_405_MOESM1_ESM.pdf]

Electronic supplementary material to:

Light-harvesting complexes of *Botryococcus braunii*

Tomas E. van den Berg[1], Bart van Oort[1], Roberta Croce[1]

[1] Biophysics of Photosynthesis, Department of Physics and Astronomy, Faculty of Sciences, Vrije Universiteit Amsterdam, 1081 HV Amsterdam, The Netherlands

Email corresponding author: r.croce@vu.nl

Journal, year: Photosynthesis Research, 2017

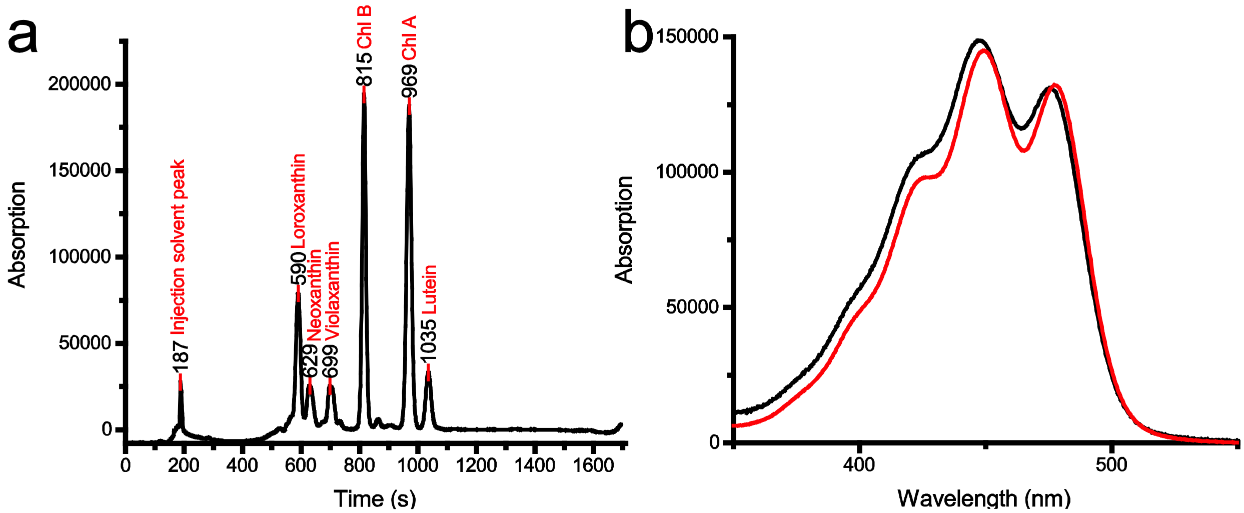

Online resource 1.

Pigment analysis of *B. braunii* trimeric LHCs. **a** HPLC chromatogram (440nm detection) **b** Absorption spectra of Loroxanthin (black) and Lutein (red) in 80% acetone normalized to the extinction coefficient.

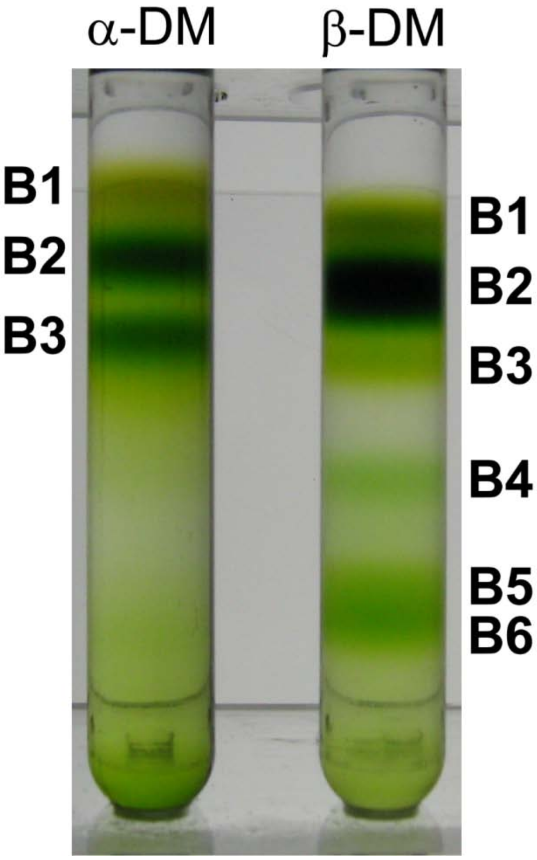

Online resource 2

Solubilization of the thylakoid membranes of *BB* with different detergents. Result of ultracentrifugation of 0.65M Sucrose gradients loaded with *BB* thylakoids solubilized by 1% α-DM or 1% β-DM. B1: free pigments, B2 Monomeric fraction, B3: Trimeric fraction, B4: PSII monomer, B5 and B6: mostly PSI with some PSII. Assignments are based on absorption and 77K fluorescence spectra, SDS-PAGE and pigment content.

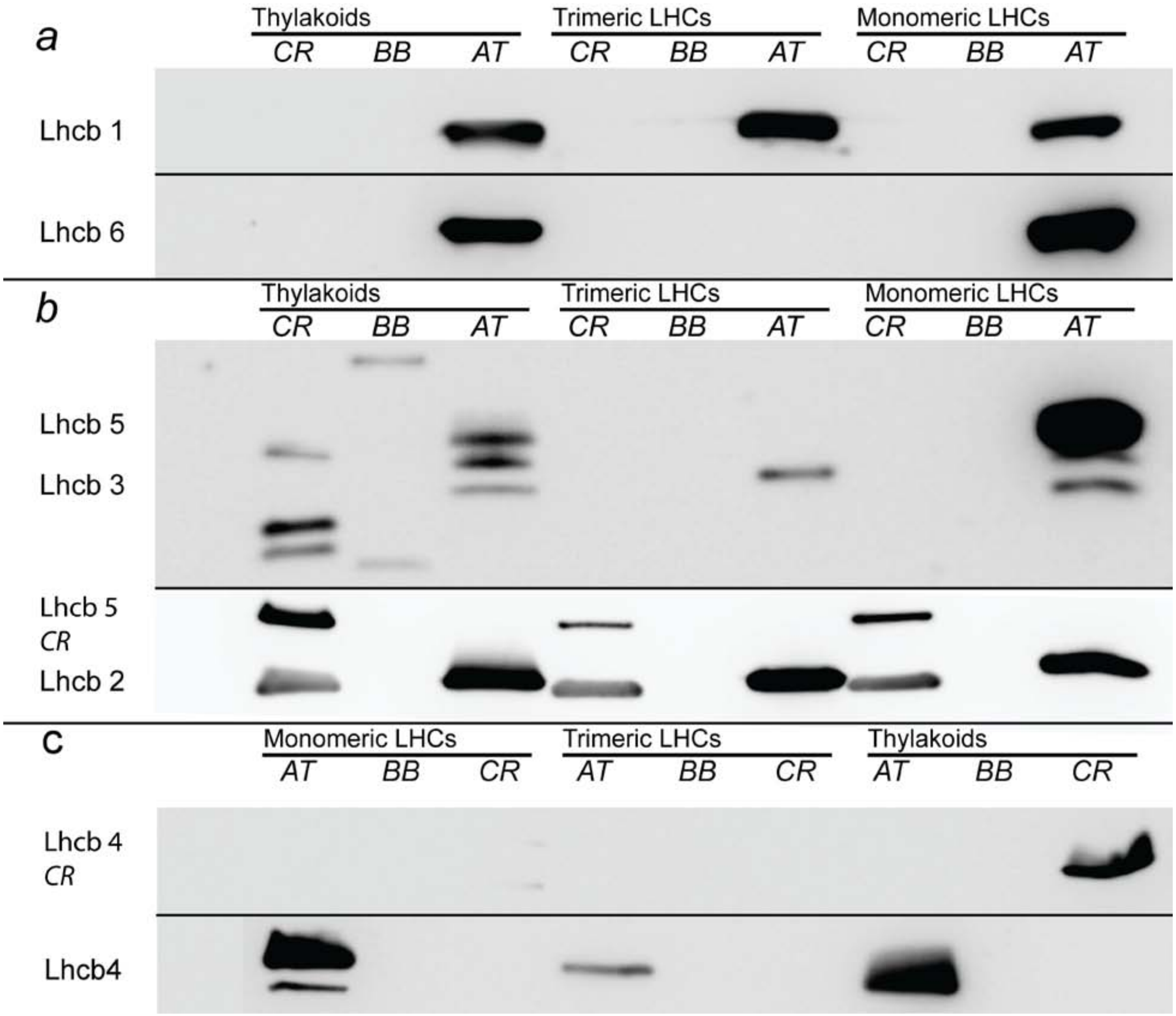

Online resource 3.

Immunoblot analyses of thylakoids, monomeric and trimeric fractions from *BB* with controls from *C. reinhardtii* (*CR*) and *A. thaliana* (*AT*) with antibodies against the main Lhc subunits (indicated on the left). Black horizontal lines indicate different membranes. No bands were detected in the *BB* fractions using *AT* Lhcb1,2,3,4,5,6 and *CR* Lhcb 4,5 antibodies

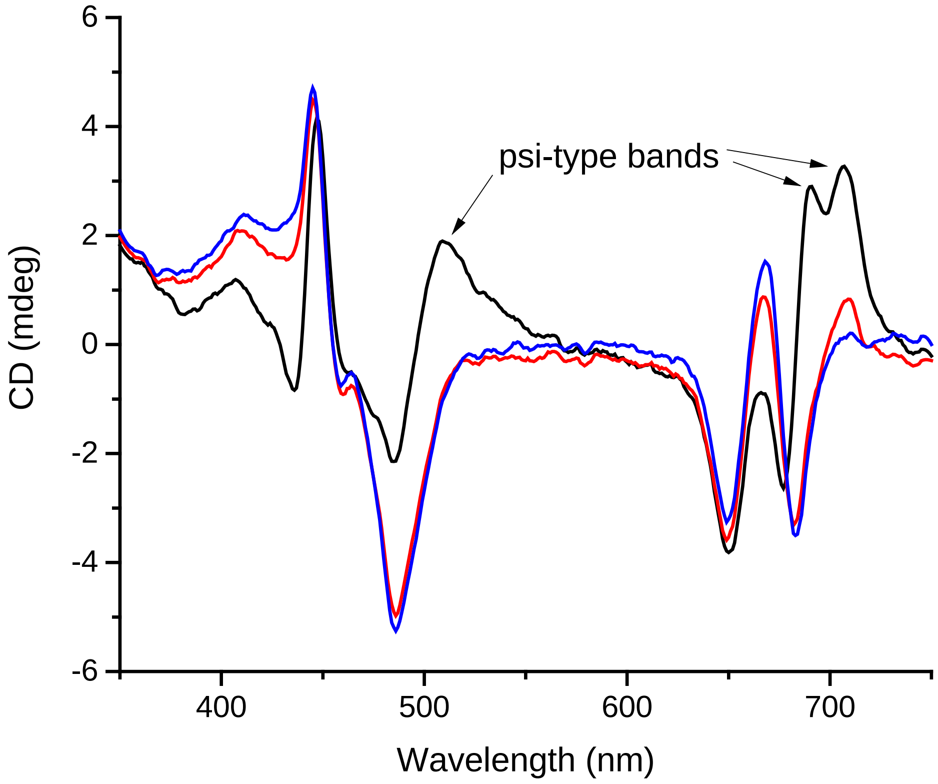

Online resource 4

CD spectrum of *B. Braunii* thylakoids, before and after solubillization. Thylakoids were measured in 20 mM Tricine-NaOH pH 7.8 at a approximate Chl concentration of 10 µg/mL at room temperature. After the measurements they were solubillized by adding 10 µL of a 10% α-DM solution (final α-DM concentration 0.1%). Black: Thylakoids, Red: within 9 min after addition of α-DM, Blue: within 39 min of solubilization at room temperature. Thylakoids have typical psi type bands and additionally a similar band structure in the carotenoid region (450-500 nm) as was observed for monomeric and trimeric complexes, confirming that this CD signal is not due to the isolation with detergent but present in the native state.

Wavelength	X^2	A_1 (%) τ=4.00 ns	A_2 (%) τ=2.12 ns	A_3 (%) τ=0.36 ns	τ_{avg} (ns)
650	0.96	66.91	20.51	12.58	3.17
655	0.965	67.09	19.63	13.29	3.17
660	1.024	67.3	18.55	14.14	3.15
665	0.982	67.72	17.85	14.43	3.16
670	1.015	68	17.71	14.3	3.17
675	1.011	68.17	18	13.83	3.18
680	0.993	68.04	18.09	13.87	3.17
685	1.022	67.88	18.55	13.57	3.18
690	1.063	67.64	18.72	13.64	3.17
695	1.014	67.52	18.99	13.49	3.17
700	0.995	66.79	18.76	14.45	3.14
705	0.995	66.71	18.1	15.19	3.13

Online resource 5

Fluorescence decay kinetics of *BB* LHC trimers. Fit results from the fluorescence decay of LHC trimers at different detection wavelengths. The traces were fitted simultaneously, while constraining the lifetimes (τ_i, see first row) to be equal at all detection wavelengths and allowing the relative amplitudes (A_i) to vary. The amplitude weighted average lifetime (τ_{avg}) was calculated as $\tau_{avg} = \sum_i A_i \tau_i / \sum_i A_i$. χ^2 is Chi square, indicating the fit quality.

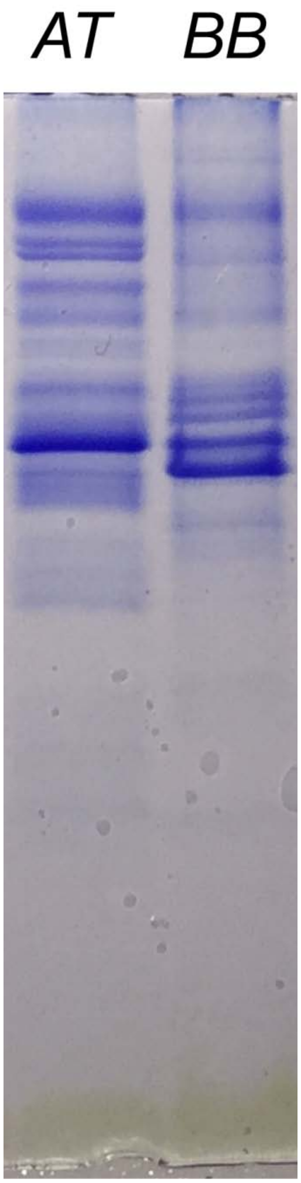

Online resource 6

SDS-PAGE loaded with thylakoid membranes of *A. thaliana* (lane 1) and *B. braunii* (lane 2). Each lane was loaded with 3 µg of Chl *a*+*b*. No large differences in protein loading are observed.
